# A new species of buffalo leech in the genus *Hirudinaria* Whitman, 1886 (Arhynchobdellida, Hirudinidae) from Thailand

**DOI:** 10.3897/zookeys.933.49314

**Published:** 2020-05-18

**Authors:** Ekgachai Jeratthitikul, Putita Jiranuntskul, Takafumi Nakano, Chirasak Sutcharit, Somsak Panha

**Affiliations:** 1 Animal Systematics and Molecular Ecology Laboratory, Department of Biology, Faculty of Science, Mahidol University, Bangkok 10400, Thailand Mahidol University Bangkok Thailand; 2 Department of Zoology, Graduate School of Science, Kyoto University, Kyoto 606-8502, Japan Kyoto University Kyoto Japan; 3 Animal Systematics Research Unit, Department of Biology, Faculty of Science, Chulalongkorn University, Bangkok 10330, Thailand Chulalongkorn University Bangkok Thailand

**Keywords:** *Hirudinaria manillensis*, Hirudinea, molecular phylogeny, new species, taxonomy, Thailand

## Abstract

*Hirudinaria manillensis* (Lesson, 1842), commonly known as the buffalo leech, shows a polymorphism of two ventral colorations. The green color morph has a plain green ventral surface and the red color morph has a brick-red ventral surface with two black submarginal stripes. Based on molecular and morphological evidence in the present study, these two color morphs were revealed as two different species. The red color morph fits well with the description of *H. manillensis*, while the green color morph showed some distinctions, and therefore is described herein as *Hirudinaria thailandica* Jeratthitikul & Panha, **sp. nov.** The new species can be distinguished from its congeners by the dark greenish or dark olive ventral surface and a round atrium with ventral insertion of ejaculatory ducts in the male reproductive organ. A phylogenetic tree based on concatenated data of COI and 28S genes supported the new species and further indicated it as a sister species to *H. bpling* Phillips, 2012.

## Introduction

“Buffalo leech” is a common name of obligatory blood-feeding ectoparasitic leeches in the genus *Hirudinaria* Whitman, 1886 and *Poecilobdella* Blanchard, 1893. Both genera belong to the subfamily Hirudinariinae Sawyer, 1986, and are characterized by the presence of a large female vaginal caecum, but are distinguishable from each other by characteristics of the female reproductive system. *Poecilobdella* species have a well-developed vaginal stalk, whereas *Hirudinaria* species are considered to lack this structure (Sawyer 1986).

The genus *Hirudinaria* is widely distributed over tropical South and Southeast Asia (Moore 1938; Lai and Chen 2010), and includes three valid species: *Hirudinaria javanica* (Wahlberg, 1856), *H. manillensis* (Lesson, 1842), and the recently described *H. bpling* Phillips, 2012. Several authors have noted that *H. manillensis* showed a color polymorphism containing two color morphs: a green color morph with darkish green on the dorsal surface and paler green on the ventral surface, and a red color morph with dark reddish brown on the dorsal surface and paler brick-red with two black submarginal stripes on the ventral surface (Moore 1927; Sawyer et al. 1998). These two color morphs occurred sympatrically in some locations, with one color morph dominant over the other in each population (Sawyer et al. 1998).

In Thailand, the first scientific report of buffalo leeches was that of Baird (1869), which introduced a new name, *Hirudo maculata* Baird, 1869, based on materials collected from Siam (now Thailand). Dequal (1917) then reported the presence of buffalo leeches in Thailand under the name *Limnatis maculosa* (Grube, 1868), based on two specimens collected from Bangkok. *Limnatis maculosa* was originally described as *Hirudo maculosa* using specimens from Singapore (Grube 1868). Both *H. maculata* and *H. maculosa* were later synonymized under *Hirudinaria manillensis* by Moore (1927). Since that time, the systematics of buffalo leeches in Thailand have not received any attention. Nearly one hundred years later, Phillips (2012) studied freshwater leeches in the southern part of Thailand and described *H. bpling* based on specimens collected from Phang Nga Province. Recently, Tubtimon et al. (2014) studied freshwater leeches collected from northeastern Thailand by investigating their morphology, COI sequences and karyotypes. These authors suggested the possibility of a new species being present in their material based on the differentiation of chromosome numbers, although the genetic divergence for these leeches was relatively low.

**Figure 1.**
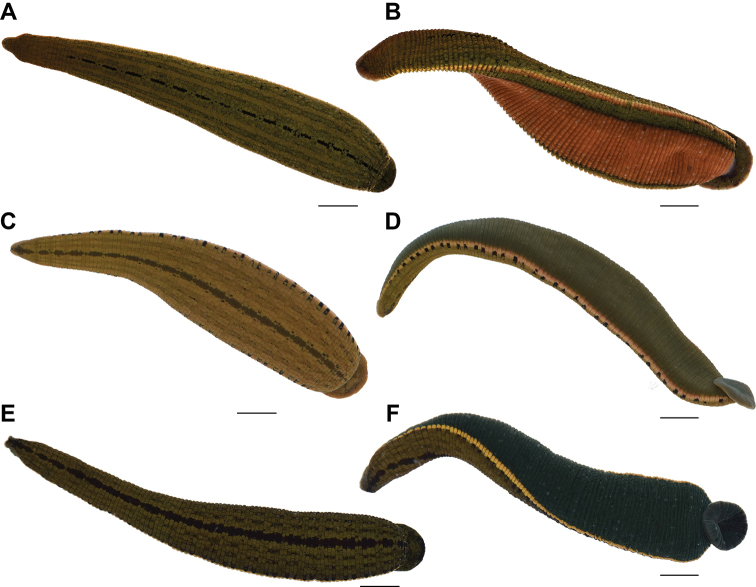
Live specimens of *Hirudinaria* species **A** dorsal and **B** ventral views of *H. manillensis* from Sakon Nakhon Province **C** dorsal and **D** ventral views of *H. bpling* from Satun Province **E** dorsal and **F** ventral views of *H. thailandica* sp. nov. MUMNH-HIR008-28 (holotype) from Chai Nat Province. Scale bar: 10 mm.

In the present study, specimens of all valid species of the genus *Hirudinaria*, including the red and green color morphs of *H. manillensis* (Fig. 1), were acquired and examined for morphological and genetic characteristics. Based on molecular and morphological evidence, the two color-morphs of *H. manillensis* were revealed as two different species. The red color morph fits well with the description of *H. manillensis*, while the green color morph showed some differences. Therefore, it is described herein as a new species.

## Materials and methods

### Specimen sampling

*Hirudinaria* leeches were collected from freshwater ponds, rice fields, and rivers in many locations in Thailand (Table 1). They were lured out of the substrate by creating gentle movements in the water. After appearing, leeches were collected by hand or with a dip net. The 2-Step Method was used for euthanasia, following AVMA Guidelines for the Euthanasia of Animals (AVMA 2013). First, animals were relaxed by the gradual addition of absolute ethanol (EtOH) to fresh water starting from approximately 5% (v/v) concentration until they became anesthetized. Then, they were moved to 70% (v/v) ethanol to complete the process. The leeches were then fixed and kept in 95% (v/v) ethanol for further external and internal morphological study. Vouchers were deposited in Mahidol University Museum of Natural History, Department of Biology, Faculty of Science, Mahidol University, Bangkok (**MUMNH**), and the Museum of Zoology, Department of Biology, Faculty of Science, Chulalongkorn University, Bangkok (**CUMZ**).

Morphological study and species identification of each specimen was based on Moore (1927), Lai and Chen (2010), Phillips (2012), and Tubtimon et al. (2014). Four measurements were taken: body length from the anterior-most point of the oral sucker to the posterior-most point of the caudal sucker (BL), maximum body width (BW), caudal sucker length (CL) and caudal sucker width (CW). Morphological examination and measurements were done under a stereo microscope (Zeiss, Stemi 305). Photographs were taken with a Nikon D5300 camera mounted with an AF-S VR Micro-Nikkor 105 mm f/2.8G IF-ED Macro Lens.

The syntypes of *Hirudo maculosa* Grube, 1868 (now synonymized with *H. manillensis*) deposited at the Museum für Naturkunde (ZMB: Zoologischen Museum Berlin), Berlin were examined to test whether the specific name *maculosa* would be resurrected: ZMB 1371, three individuals-the largest syntype (BL 57.95 mm, BW 7.66 mm; CL 8.27 mm, CW 8.66 mm), dissected; the next largest (BL 28.99 mm, BW 3.27 mm; CL 3.15 mm, CW 3.41 mm); and the smallest (BL 19.31 mm, BW 3.45 mm; CL 2.41 mm, CW 2.59 mm).

**Table 1.** Locality with geographic coordinates and GenBank accession numbers for specimens used in phylogenetic analysis.

Species	Voucher ID	Locality	Coordinates	COI	28S
*Hirudinaria manillensis* (Lesson, 1842)	MUMNH-HIR014-01	Thailand, Nong Khai Province, Phon Phisai District, Swamp near Chum Chang	17°58.47'N, 103°05.06'E	MN882682	MN882665
	MUMNH-HIR015-16	Thailand, Bueng Kan Province, Seka District, paddy fields near Sang	17°50.13'N, 103°56.40'E	MN882683	MN882666
	MUMNH-HIR012-01	Thailand, Phitsanulok Province, Wat Bot District, daddy field near Tho Thae	16°56.99'N, 100°19.73'E	MN882684	MN882667
	CUMZ 3405 (NK1)	Thailand, Nakhon Phanom Province, Na Wa District, Ban Donsala, Nong Kok	17°34.45'N, 104°07.31'E	MN882685	MN882668
	CUMZ 3406 (NK2)	Thailand, Nakhon Phanom Province, Si Songkhram District, Ban Don Ma Chang	17°36.88'N, 104°08.36'E	MN882686	MN882669
*Hirudinaria javanica* (Wahlberg, 1856)	MUMNH-HIR013-01	Thailand, Nakhon Phanom Province, Si Songkhram District, Huai Khon Reservoir	17°36.93'N, 104°17.57'E	MN882687	MN882670
	CUMZ 3422 (MD2)	Thailand, Mukdahan Province, Khamcha-I District, paddy fields near Ban Nonghai	16°34.89'N, 104°29.48'E	MN882688	MN882671
	CUMZ 3405(NK3)	Thailand, Nakhon Phanom Province, Na Wa District, Ban Donsala, Nong Kok	17°34.45'N, 104°07.31'E	MN882689	MN882672
*Hirudinaria bpling* Phillips, 2012	MUMNH-HIR016-04 MUMNH-HIR016-05	Thailand, Phang Nga Province, Kapong District, swamp near 3004 Rd.	8°46.08'N, 98°27.33'E	MN882690 MN882691	MN882673 MN882674
	MUMNH-HIR016-02	Thailand, Satun Province, Mueang District, pond near Nong Plak Phra Ya	6°44.48'N, 100°02.47'E	MN882692	MN882675
*Hirudinaria thailandica* sp. nov.	MUMNH-HIR008-28 (holotype)	Thailand, Chai Nat Province, Mueang District, Lotus pond near Ban Kluai	15°10.65'N, 100°08.70'E	MN882693	MN882676
	MUMNH-HIR003-08	Thailand, Phrae Province, Sung Men District, unnamed stream near Ban Kwang	18°04.26'N, 100°11.26'E	MN882694	MN882677
	MUMNH-HIR009-02	Thailand, Buriram Province, Krasang District, Chi River at Nong Teng	14°52.51'N, 103°22.72'E	MN882695	MN882678
	MUMNH-HIR001-01	Thailand, Mukdahan Province, Wan Yai District Chanot Stream at Wan Yai	16°43.76'N, 104°43.77'E	MN882696	MN882679
	MUMNH-HIR004-01	Thailand, Nakhon Phanom Province, Tha Uthen District, Songkhram River	17°39.10'N, 104°27.85'E	MN882697	MN882680
	MUMNH-HIR010-04	Thailand Ubon Ratchathani Province, Khemmarat District, Huai Na Muang stream	16°01.75'N, 105°15.91'E	MN882698	MN882681
*Hirudo verbena* Carena, 1820	–	–	–	HQ691223 ^a^	HQ691219 ^a^
*Aliolimnatis oligodonta* (Johansson, 1913)	–	Tanzania	–	GQ368739 ^b^	GQ368762 ^b^
*Poecilobdella nanjingensis* Yang, 1996	KUZ Z1743	Neidong Forest Trail, Wulai District, New Taipei City, Taiwan	24°49.80'N, 121°31.92'E	LC145739 ^c^	LC145738 ^c^

^a^Novo et al. (2011); ^b^Phillips and Siddall (2009); ^c^Nakano and Lai (2016)

### Molecular analysis

The total genomic DNA was extracted from the body-wall muscles to avoid contamination from any host DNA by using a NucleoSpin Tissue kit (MACHEREY-NAGEL), following the manufacturer’s standard protocol. Fragments of mitochondrial cytochrome c oxidase subunit I (COI) gene and the nuclear 28S ribosomal RNA large subunit gene (28S) were amplified and used to estimate molecular phylogeny. Primers used for polymerase chain reaction (PCR) and sequencing are presented in Table 2. For COI, two primer sets were used to amplify the 1266 bp sequence: 1) LCO1490hiru and HCO2198hiru and 2) LCOinHiru and HCO-out (Nakano 2012); two other primer sets were used for the approximately 1200 bp sequence of the 28S gene: 1) 28F2-2 and 28R2 (Passamaneck et al. 2004) and 2) 28F2-3 and 28R3 (Jördens et al. 2004; Passamaneck et al. 2004). The PCR amplification was conducted in a final volume of 20 µl containing 1 µl of each primer (10 mM), 10 ng of the extracted genomic DNA, 10 µl of EmeraldAmp PCR Master Mix (TAKARA BIO INC.) and distilled water up to 20 μL total volume. Each PCR reaction was performed using a T100 thermal cycler (BIO-RAD). The thermal cycling was started at 94 °C for 3 min, followed by 35 cycles of 94 °C for 30 sec, 42–52 °C (depending on gene and primer set) for 60 sec, extension at 72 °C for 90 sec, then followed by a final 72 °C for 5 min. All PCR products were purified by PEG precipitation methods and then sent to Bio Basic Inc., Canada for bi-directional sequencing on an automated sequencer (ABI prism 3730XL). Nucleotide sequences were deposited in the GenBank database under GenBank submission numbers: MN882665–MN882698 (Table 1).

**Table 2.** Sequences of primers used for PCR amplification and sequencing gene fragments in this study. Those marked with ‘*’ were specifically modified for *Hirudinaria* species (F = Forward, R = Reverse).

Genes	Amplicon length (bp)	Primer name	Primer sequence (5’ to 3’)	Reference
**COI**				
1	720	LCO1490hiru (F)	ATT CTA CTA GTC ATA AAG ATA TTG G	This study
		HCO2198hiru (R)	AAA ATC AAA ATA TAT ACT TCT GGA TG	This study
2	805	LCOinHiru (F)	GAA ATG AGC GAG TCC TTT ATT TG	This study
		HCO-out* (F)	TCT GGA TAG TCT GAA TAT CG	Nakano (2012)
**28S**				
1	802	28F2-2 (F)	GCA GAA CTG GCG CTG AGG GAT GAA C	Passamaneck et al. (2004)
		28R2 (R)	GAG GCT GTK CAC CTT GGA GAC CTG CTG CG	Passamaneck et al. (2004)
2	824	28F2-3 (F)	ATC GAA AGG GAA TCG GGT TAA TAT TCC	Jördens et al. (2004)
		28R3 (R)	GAT GAC GAG GCA TTT GGC TAC C	Passamaneck et al. (2004)

Sequences were aligned and edited using ClustalW as implemented in MEGA7 (Kumar et al. 2016). The concatenated dataset of COI + 28S genes was used for phylogenetic tree reconstruction using maximum likelihood (ML) and Bayesian inference (BI) approaches, and with *Poecilobdella nanjingensis* Yang, 1996, *Hirudo verbena* Carena, 1820, and *Aliolimnatis oligodonta* (Johansson, 1913) as outgroups. The ML analysis was conducted using 1000 ML bootstrap replications and GTRGAMMA as the model for all gene partitions in the program RAxML v.8.2.10 (Stamatakis 2014). The BI analysis was performed in the program MrBayes 3.2.6 (Ronquist et al. 2012) with the Markov chain Monte Carlo analysis (MCMC) in two parallel runs and with four chains each. The best-fit evolution models based on the Akaike Information Criterion (AIC: Akaike 1974) as suggested by the program KAKUSAN4 (Tanabe 2011) were GTR+G for both COI and 28S. Markov chains were run using random starting tree for 10 million generations and tree sampling every 1000^th^ generation. The first 25% of obtained trees were discarded as burn-in. The remaining trees were used to estimate the consensus tree topology, bipartition posterior probability (bpp) and branch length. The effective sample size value sampled from the MCMC analysis was greater than 8000 for all parameters. Both Ml and BI were run through the on-line CIPRES Science Gateway (Miller et al. 2010). Nodes with 0.95 or higher bpp and/or 70% or higher bootstrap value were regarded as sufficiently supported (Huelsenbeck and Hillis 1993; Larget and Simon 1999).

Genetic divergences based on the COI sequences were also calculated to depict evolutionary divergence between *Hirudinaria* species using uncorrected p-distances as implemented in MEGA7 (Kumar et al. 2016).

## Taxonomy

### Family Hirudinidae Whitman, 1886

Subfamily Hirudinariinae Sawyer, 1986

Genus *Hirudinaria* Whitman, 1886

#### Hirudinaria thailandica

Taxon classificationAnimaliaArhynchobdellidaHirudinidae

Jeratthitikul & Panha sp. nov.

68671F97-9A37-5C69-A981-15D6944A533A

http://zoobank.org/47A4B84B-DDC2-4E7F-9B9A-FBA9FD3C6D0A

Figs 1E, F , 2 , 3

##### Type materials.

***Holotype*.** Thailand • Chai Nat Province, Mueang Chai Nat District, Ban Kluai Subdistrict; 15°10.65'N, 100°08.70'E; 17 Mar. 2018; ASME members leg.; Lotus pond; MUMNH-HIR008-28; dissected (BL 111.70 mm, BW 15.50 mm; CL 12.45 mm, CW 12.85 mm). ***Paratype*.** Thailand • 2 specimens; same collection data as for holotype; MUMNH-HIR008-01; dissected (BL 129.15 mm, BW 13.35 mm; CL 11.90 mm, CW 11.95 mm), MUMNH-HIR008-02; dissected (BL 117.25 mm, BW 13.05 mm; CL 11.70 mm, CW 12.30 mm).

##### Other materials.

Thailand • 25 specimens; same collection data as for holotype; MUMNH-HIR008-03 to HIR008-27 • 1 specimen; Mukdahan Province, Wan Yai District, Wan Yai Subdistrict, Chanot Stream; 16°43.76'N, 104°43.77'E; 1 Apr. 2018; ASME members leg.; MUMNH-HIR001-01 • 1 specimen; Nakhon Phanom Province, Tha Uthen District, Songkhram River; 10 Apr. 2018; E. Jeratthitikul leg.; MUMNH-HIR004-01 • 8 specimens; Phrae Province, Sung Men District, Ban Kwang Subdistrict; 18°04.26'N, 100°11.26'E; 13 May. 2018; Local peoples leg.; MUMNH-HIR003-01 to HIR003-08 • 3 specimens; Buriram Province, Krasang District, Nong Teng Subdistrict, Chi River; 14°52.51'N, 103°22.72'E; 18 Sep. 2018; E. Jeratthitikul and C. Sutcharit leg.; MUMNH-HIR009-01 to HIR009-03 • 4 specimens; Ubon Ratchathani Province, Khemmarat District, Huai Na Muang stream; 16°01.75'N, 105°15.91'E; 12 May. 2018; E. Jeratthitikul leg.; MUMNH-HIR010-01 to HIR010-04.

**Figure 2.**
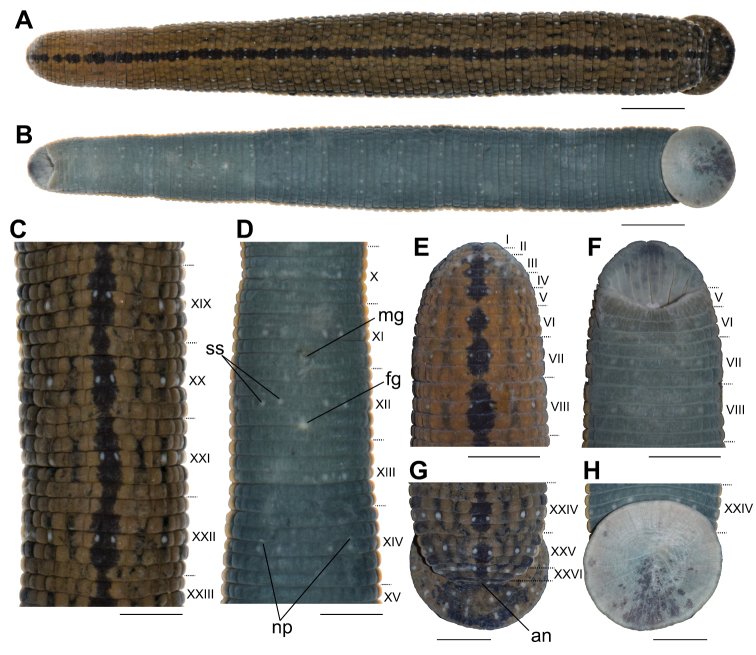
External morphology of the holotype of *Hirudinaria thailandica* sp. nov. in preserved state **A** dorsal and **B** ventral views of the whole body **C** dorsal views of somites XIX–XXIII **D** ventral views of somites X–XV **E** dorsal and **F** ventral views of somites I–VIII **G** dorsal and **H** ventral views of somites XXIV–XXVII and caudal sucker. Abbreviations: an, anus; fg, female gonopore; mg, male gonopore; np, nephridiopores; and ss, sensillae. Scale bar: 10 mm (**A, B**), 5 mm (**C–H**).

##### Diagnosis.

In life, dorsal surface dark brown to green. Lateral spots on annuli b2 and b5, black, squared, not prominent, reduced to small spots on anterior somites. Ventral surface green to dark greenish or dark olive. Male gonopore in somite XI b5/b6. Female gonopore in somite XII b5/b6. Gonopores separated by 5 annuli. Atrium of male reproductive organ moderate-sized, bulbous, rather round. Penis sheath short. Ejaculatory ducts ventrally inserted into middle of atrium. Common oviducts open into the female bursa near the insertion point of vaginal caecum to female bursa.

**Figure 3.**
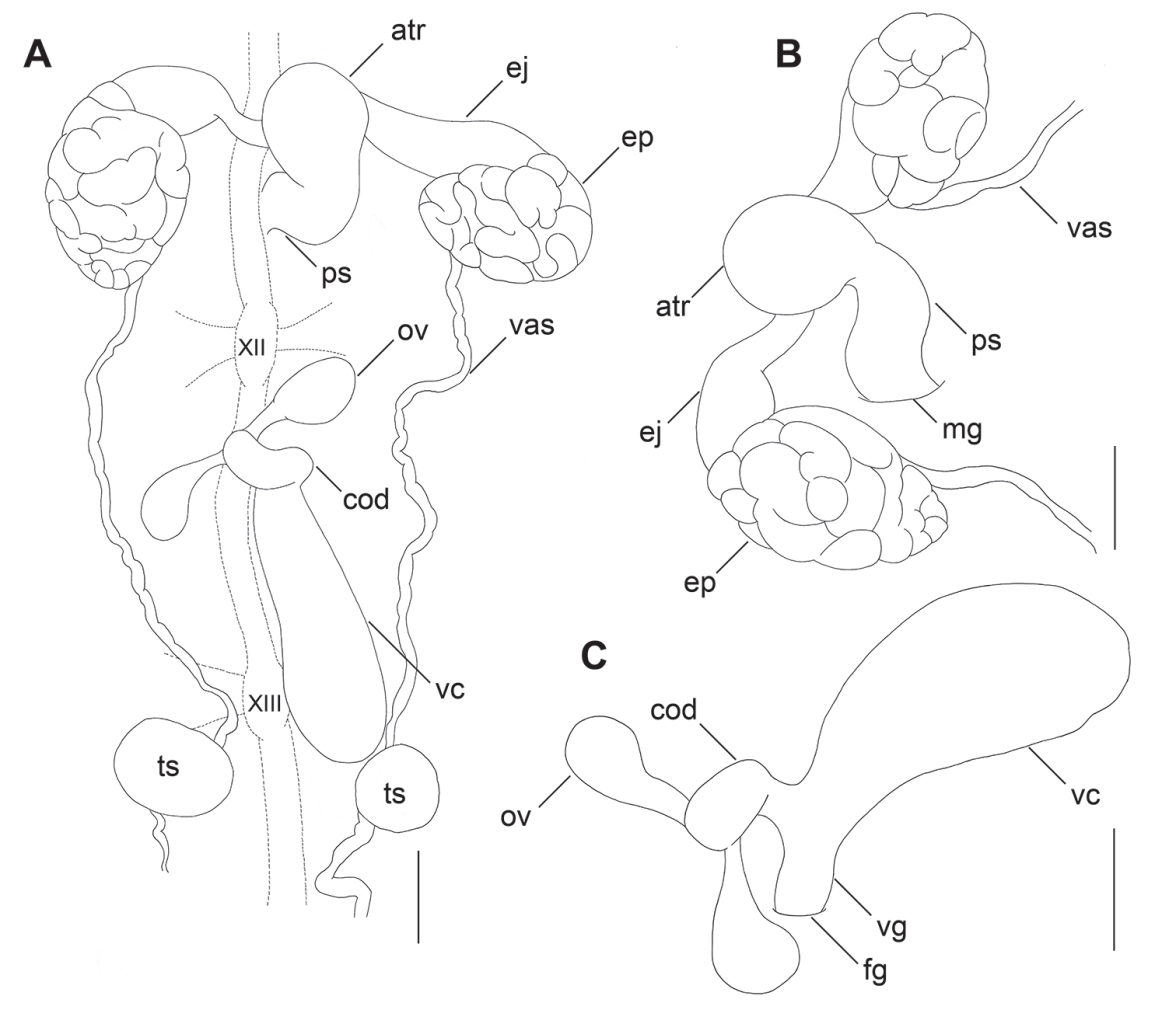
Illustration of reproductive system of the holotype of *Hirudinaria thailandica* sp. nov. **A** dorsal view of male and female reproductive organs including positions of ganglia XII and XIII of the ventral nervous system. Lateral views of **B** male and **C** female reproductive organs. Scale bar: 2 mm. Abbreviations: atr, male atrium; cod, common oviduct; ej, ejaculatory bulb; ep, epididymis; fg, female gonopore; mg, male gonopore; ov, ovary; ps, penis sheath; ts, testisac. vas, vas deferens; vc, vaginal caecum; and vg, vagina.

##### Description of holotype.

Body firm, muscular, with constant width posteriorly. In life, dorsal surface dark brown to green in background color. Dorsal median line black, distinct, somewhat narrow on annuli b1 and b6 of each mid body somite, and very narrow or broken between somites V and VI, VI and VII, and VII and VIII. Two longitudinal, inconspicuous, and broken stripes on each side of median stripe, each stripe present in b2–b5 and absent in b1 and b6 of each mid body somite. Lateral spots on annuli b2 and b5, black, squared, not prominent, reduced to small spots on some anterior somites. Ventral surface green to dark greenish or dark olive, with narrow marginal pale-yellow stripe. Color faded in preservative, except the dorsal median line and other dorsal markers.

Number of annuli 103. Somites I–III uniannulate. Somites IV and V biannulate, (a1+a2)=a3. Somite VI dorsally triannulate, a1=a2<a3; ventrally biannulate, (a1+a2)=a3. Somite VII triannulate, a1=a2<a3. Somite VIII quadrannulate, a1>a2=b5>b6. Somites IX–XXIII quinquannulate, b1=b2=a2=b5=b6. Somite XXIV quadrannulate, b1=b2=a2=a3. Somite XXV being last complete annulus ventrally; triannulate, a1=a2=a3. Somite XXVI biannulate, (a1+a2)>a3. Somite XXVII biannulate, (a1+a2)>a3. Anus on somite XXVII a3. Clitellum between X b5 and XIII a2.

Eyespots 5 pairs, arranged dorsally in parabolic arch: first pair anterior on somite II, remaining eyespots situated laterally; second pair on somite III; third pair on somite IV (a1+a2); fourth pair on somite V (a1+a2); and fifth pair on somite VI a2. Sensillae on annulus a2 of complete somites; 3 bilateral rows dorsally, one proximal to midline, 2 paired distal to midline; and 2 bilateral rows ventrally. Nephridiopores 17 pairs, positioned on ventral surface, situated bilaterally at posterior margin of VIII a1, and at posterior margin of b2 of somites IX to XXIV.

Oral sucker, dorsal lip prominent, with several furrows. One median longitudinal furrow on ventral surface deep, extending from margin of lip to pharynx. Two deep furrows on each side, and one or two shallow furrows between deep furrows. Three jaws in oral cavity, 1 dorsal and 2 ventrolateral. Small salivary papillae circular, placed along denticular ridge. Large salivary papillae circular to ovate; irregularly placed laterally on each jaw; minimum of 30 visible on one side of right ventro-lateral jaw. Teeth on median jaw 105–125 per jaw. Pharynx muscular and tubular reaching to somite IX b2/a2. Crop reaching to somite XIX b5. Crop caeca10 pairs: Intestine tubular, acaecate, extending from somite XIX b6 to somite XXIII. Rectum thin-walled, tubular, extending from somite XXIII to somite XXVII. CL 11.9 mm, 13.0 mm wide, round, with eight rays of sensillae on dorsal surface radiating out from center.

Male gonopore in somite XI b5/b6. Female gonopore in somite XII b5/b6. Gonopores separated by 5 annuli. Nerve cord passing along the left side of reproductive system. Male reproductive system in somites XI and XII. Atrium moderate-sized, bulbous, rather round. Penis sheath short, recurved anteriorly to atrium. Epididymes round, paired, lateral to atrium. Ejaculatory bulbs present, muscular, and thick. Ejaculatory ducts short, ventrally inserted into middle of atrium. Testisacs 11 pairs. Female reproductive system in somites XII and XIII.

Ovaries paired, small, droplet-shaped. Oviduct paired, narrow, short, join to form common oviduct. Common oviduct moderate in length, slightly bent distally, then descending to vagina. Albumin gland at point of intersection of two oviducts and common oviduct. Common oviduct opens into female bursa near the insertion point of vaginal caecum to female bursa. Vaginal caecum elongated, two times as long as wide at widest point. Vaginal caecum anteroventral end connected to female bursa.

##### Etymology.

The specific name “*thailandica*” refers to the country in which specimens of the new species were collected.

##### Distribution.

**Figure 4.**
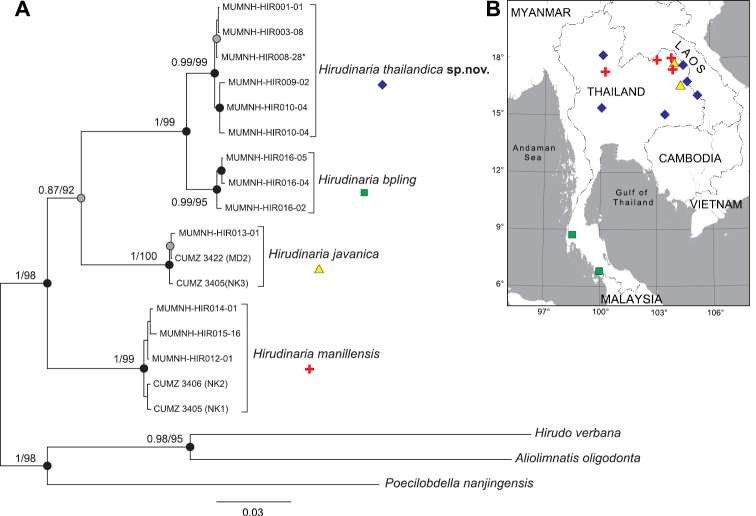
Phylogenetic analysis of *Hirudinaria* species and related taxa **A** bayesian inference tree based on 2533 bp alignment dataset of nuclear 28S rRNA and mitochondrial COI genes. Numbers at nodes indicate bootstrap values from maximum likelihood (ML) and bpp from Bayesian inference analysis (BI). Nodes with black circles are sufficiently supported by both BI and ML, while nodes with grey circles are supported only by ML**B** map showing the sampling locations for the specimens used for DNA sequence analysis.

This species is known from several river basins in Thailand, including the Chao Phraya and the Middle Mekong river basins (Fig. 4B).

### Molecular phylogenies and genetic divergence

Phylogenetic trees estimated by ML and BI gave equivalent topologies. Therefore, only a BI tree is shown in Fig. 4A. The monophyly of the genus *Hirudinaria* and of each analyzed *Hirudinaria* species was strongly supported (ML bootstrap values of 95–100% and a BIbpp of 0.99–1.0). Evolutionary relationships among *Hirudinaria* species and related taxa were highly supported for all major nodes (ML bootstrap values of 92–98% and a BIbpp of 1.0), except the node of *H. thailandica*, *H. bpling*, and *H. javanica*, where BI gave a relatively low bpp of 0.87. Within the *Hirudinaria* clade, *H. manillensis* was placed at the basal part of the tree. The next was *H. javanica*, whereas, *H. thailandica* was clustered as a sister clade to *H. bpling*.

The average interspecific divergence based on uncorrected p-distances for COI sequences between *H. thailandica* and other congeners was 9.21% (4.51%–12.12%; Table 4). That between *H. thailandica* and its sister, *H. bpling* was 4.51%. Average intraspecific divergences within each *Hirudinaria* species were low to moderate, ranging from 0.40% in *H. javanica* to 0.80% in *H. thailandica*.

## Discussion

*Hirudinaria thailandica* belongs in the genus *Hirudinaria* based on the large vaginal caecum and the lack of a vaginal stalk (Moore 1927; Sawyer 1986). It also diﬀers from the other three congeners in this genus by a combination of characteristics as summarized in Table 3. The morphology of the new species is most similar to *H. bpling*. In addition, the phylogenetic tree also revealed them as sister taxa (Fig. 4A). They share a general pattern of a greenish coloration in external features. However, they are distinguishable from each other by several external and internal morphological features. *Hirudinaria thailandica* differs from *H. bpling* by possessing a series of faint lateral spots, rather than a series of completely black, large, and prominent lateral spots; a dark greenish or dark olive ventral surface rather than a lighter green ventral surface; ejaculatory ducts that insert into the midventral part of the male atrium, rather than the anteroventral part of the male atrium; and in that the common oviduct opens into the female bursa near the insertion point between the vaginal caecum and the female bursa rather than directly opening into the dorsal portion of the vaginal caecum. Moreover, their distribution ranges are limited to different river basins (Fig. 4B). *Hirudinaria bpling* is distributed in western coastal rivers that drain into the Andaman Sea and Peninsular Malaysia (Chong et al. 2014), while *H. thailandica* is restricted to the Chao Phraya and Mekong basins.

**Table 3.** Morphological comparison among the known species of the genus *Hirudinaria*.

Characters	*H. manillensis* (Lesson, 1842)	*H. thailandica* sp. nov.	*H. bpling* Phillips, 2012	*H. javanica* (Wahlberg, 1856)
Ventral surface color	brick-red to brown	green to dark greenish or dark olive	dark green	green
Submarginal stripe on ventral surface	present	absent	absent	absent
Number of annuli between gonopores	5	5	5	7
Atrium	bulbous	long	bulbous	short
Ejaculatory ducts	inserted anteroventrally into atrium	inserted medially into atrium	inserted anteroventrally into atrium	inserted laterally into atrium

**Table 4.** Average interspecific genetic divergence (uncorrected p-distance: %±SE) matrix for the 658 bp barcoding region of COI gene between *Hirudinaria* species (below diagonal) and average intraspecific distances within each taxon (in bold).

**Taxa**	**1.**	**2.**	**3.**	**4.**
1. *Hirudinaria manillensis*	**0.66±0.22**			
2. *Hirudinaria javanica*	10.16 ±1.10	**0.40±0.20**		
3. *Hirudinaria bpling*	12.75±1.23	11.41±1.11	**0.50±0.21**	
4. *Hirudinaria thailandica* sp. nov.	12.12±1.18	11.01±1.23	4.51±0.74	**0.80±0.23**

The present new species can be clearly distinguished from the Singaporean *Hirudo maculosa* by its male ejaculatory ducts reaching to the midventral part of the male atrium, while the latter possesses ejaculatory ducts running toward the anteroventral part of the male atrium. In fact, morphological characteristics of *H. maculosa* resemble those of the recently described, *Hirudinaria bpling*, by Phillips (2012). However, DNA examination using fresh materials of *Hirudo maculosa* collected from the type locality is necessary before any further taxonomic conclusions can be made.

Genital characteristics of *H. thailandica* are most similar to that of *H. manillensis*. This is the reason that some authors included this species as a variant of *H. manillensis* (Moore 1927; Sawyer 1986; Tubtimon et al. 2014). However, their external morphologies are clearly distinct, especially in the coloration of the ventral surface. The new species possesses a dark greenish ventral surface, while *H. manillensis* has a brick-red ventral surface bordered by two additional black submarginal stripes (Fig. 1). The coloration and pattern of the ventral surface is one of the most helpful characters in identifying leech species, such as in the genera *Hirudo* Linnæus (Trontelj and Utevsky 2012) and *Hirudinaria*.

## Supplementary Material

XML Treatment for Hirudinaria thailandica
